# Comprehensive analysis of the potential cuproptosis-related biomarker LIAS that regulates prognosis and immunotherapy of pan-cancers

**DOI:** 10.3389/fonc.2022.952129

**Published:** 2022-08-02

**Authors:** Yuan Cai, Qingchun He, Wei Liu, Qiuju Liang, Bi Peng, Jianbo Li, Wenqin Zhang, Fanhua Kang, Qianhui Hong, Yuanliang Yan, Jinwu Peng, Zhijie Xu, Ning Bai

**Affiliations:** ^1^ Department of Pathology, Xiangya Hospital, Central South University, Changsha, China; ^2^ Department of Pathology, Xiangya Changde Hospital, Changde, China; ^3^ Department of Emergency, Xiangya Hospital, Central South University, Changsha, China; ^4^ Department of Emergency, Xiangya Changde Hospital, Changde, China; ^5^ Department of Orthopedic Surgery, The Second Hospital University of South China, Hengyang, China; ^6^ Department of Pharmacy, Xiangya Hospital, Central South University, Changsha, China; ^7^ National Clinical Research Center for Geriatric Disorders, Xiangya Hospital, Central South University, Changsha, China; ^8^ Department of General Surgery, Xiangya Hospital, Central South University, Changsha, China

**Keywords:** cuproptosis, LIAS, immunity, prognosis, pan-cancer

## Abstract

Lipoic acid synthetase (LIAS) has been demonstrated to play a crucial role in the progression of cancer. Exploring the underlying mechanisms and biological functions of LIAS could have potential therapeutic guidance for cancer treatment. Our study has explored the expression levels and prognostic values of LIAS in pan-cancer through several bioinformatics platforms, including TIMER2.0, Gene Expression Profiling Interactive Analysis, version 2 (GEPIA2.0), and Human Protein Atlas (HPA). We found that a high LIAS expression was related to the good prognosis in patients with kidney renal clear cell carcinoma (KIRC), rectum adenocarcinoma (READ), breast cancer, and ovarian cancer. Inversely, a high LIAS expression showed unfavorable prognosis in lung cancer patients. In addition, the genetic alteration, methylation levels, and immune analysis of LIAS in pan-cancer have been evaluated. To elucidate the underlying molecular mechanism of LIAS, we conduct the single-cell sequencing to implicate that LIAS expression was related to hypoxia, angiogenesis, and DNA repair. Thus, these comprehensive pan-cancer analyses have conveyed that LIAS could be potentially significant in the progression of various cancers. Moreover, the LIAS expression could predict the efficacy of immunotherapy in cancer patients.

## Introduction

The aberrant alternations of genes may affect the cancer pathogenesis and therapeutic response, resulting in cancer progression. Oncogenes and cancer suppressor genes exert opposite effects on the pathological process of cancers (1, 2). The exploration of cancer-associated genes and signaling pathways could be beneficial to the diagnose and clinical therapy of most cancers.

Lipoic acid synthetase (LIAS) has been found to participate in the synthesis of mitochondria-associated metabolic enzymes, involved in energy metabolism and antioxidant response (3). Mutations in LIAS caused the defect in mitochondrial energy metabolism (4). Several studies over the past decade have delineated that the genetic mutations in LIAS might lead to complex metabolic diseases. A newborn with homozygous mutation in the LIAS gene was found to suffer from epilepsy, lactic acidosis, and glycine buildup (5). Although LIAS alterations have been detected in several mitochondrial dysfunction diseases, including cancers, its underlying mechanisms and potential biological functions have not been well investigated.

In this article, we investigated the regulation functions of LIAS in various kinds of cancers by a series of bioinformatics platforms (**Table 1**). In our study, we compared the expression profiles of LIAS between tumor tissues and the corresponding normal tissues. Also, survival analysis, methylation levels, and the roles in immune regulation were also evaluated in this study. The above findings indicated that LIAS was significantly related to the cancer pathogenesis and immune response.

**Table 1:** The bioinformatics databases that are applied for exploring regulation functions of LIAS.

Databases	URL	References
TIMER2.0	http://timer.cistrome.org/	(6)
GEPIA2.0	http://gepia2.cancer-pku.cn/#index	(7)
TNMplot	https://tnmplot.com/analysis/	(8)
UALCAN	http://ualcan.path.uab.edu/analysisprot.html	(9)
HPA	http://www.proteinatlas.org	(10)
cBioPortal	https://www.cbioportal.org/	(11)
CancerSEA	http://biocc.hrbmu.edu.cn/CancerSEA/home.jsp	(12)
BioGRID	https://thebiogrid.org	(13)
Kaplan-Meier plotter	http://kmplot.com/analysis/	(14)
TIDE	http://tide.dfci.harvard.edu/	(15)

## Materials and methods

### The expression evaluations

We applied TIMER2.0 (6), Gene Expression Profiling Interactive Analysis, version 2 (GEPIA2.0) (7), and TNMplot (8) to analyze the expression profiles of LIAS between the tumor tissues and the corresponding normal tissues. The GEPIA2.0 database was applied to conduct the analysis based on TCGA and genotype-tissue expression dataset (GTEx) samples. The screening criteria in GEPIA2.0 were as follows: p < 0.05 and the cutoff of |Log_2_FC| was 0.1. At the same time, we used the GEPIA2.0 database to analyze the relationship between the LIAS expression and pathological stages across TCGA cancers. The UALCAN platform (9) was employed to investigate the methylation levels and protein levels of LIAS in TCGA cancers. The Human Protein Atlas (HPA) (10) database was used to analyze the protein levels of LIAS in tumor groups and normal groups.

### Genetic alteration evaluations

The cBioPortal platform (11) was employed to evaluate the genetic alteration of LIAS across TCGA cancers, such as mutation, structural variant, amplification, and deep deletion. Also, cBioPortal was used to conduct the survival analysis of cancers with or without LIAS genetic alteration, including overall survival (OS), disease-specific survival (DSS), disease-free survival (DFS), and progression-free survival (PFS).

### Immune evaluations

TIMER2.0 (6) was used to analyze the relationship between LIAS expression and immune infiltrating cells, including B cells, cancer-associated fibroblast (CAF), T-cell CD8+ cells, dendritic cells (DC), macrophage, T-cell regulatory (Tregs), neutrophil, NK cells, and monocytes. Meanwhile, using TIDE (15), we investigated the correlation between the LIAS expression and immune checkpoint blockade response in bladder cancer and melanoma patients.

### Single-cell sequencing analysis

The CancerSEA tool (12) was employed to analyze the biological functions of cancer-related genes at the single-cell level. The heatmap indicated the roles of LIAS expression on biological functions which were downloaded from the CancerSEA database. The machine learning technique, t-distributed stochastic neighbor embedding (t-SNE), was used to identify the expression distribution of LIAS in cancer. t-SNE was a useful technique for analyzing the high-dimensional data with minimal tuning of the parameters (16).

### LIAS-associated gene enrichment analysis

The BioGRID (13) tool was employed for the LIAS-associated protein network analysis. Also, we applied the GEPIA2.0 database to explore the top 100 LIAS-related genes in TCGA pan-cancer. Furthermore, the evaluation of Kyoto Encyclopedia of Genes and Genomes (KEGG) signaling pathways regulated by the LIAS-related genes was conducted by Xiantao Xueshu (https://www.xiantao.love/products).

### Survival analysis

The GEPIA 2.0 database was employed to obtain the survival significance of LIAS in cancer patients, including OS and DFS. Also, the Kaplan–Meier plotter (14) was used to conduct the survival analysis of LIAS in breast cancer, ovarian cancer, and lung cancer. P < 0.05 was considered to be significantly important. Moreover, the Student’s t-test, Cox regression analysis, and linear regression analysis were conducted.

## Results

### The expression levels of LIAS in pan-cancer

**Figure 1:**
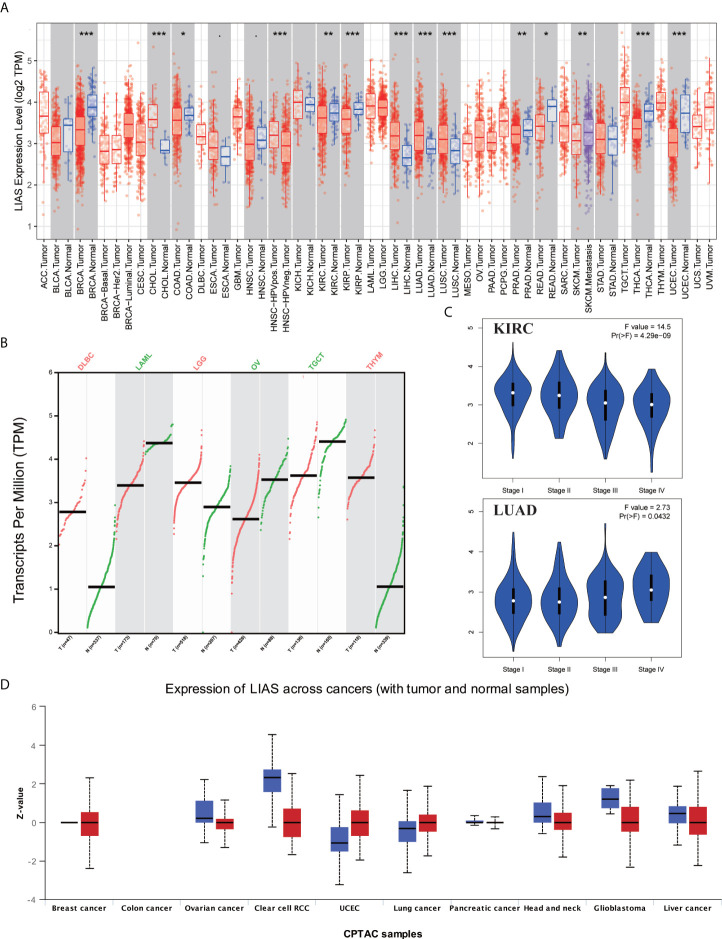
The expression levels of LIAS in pan-cancer. **(A)** TIMER2.0 showed the expression of LIAS across TCGA cancers and the corresponding normal tissues. ***p < 0.001; **p < 0.01; *p < 0.05. **(B)** GEPIA2.0 depicted the LIAS expression in the tumor group and normal group. **(C)** GEPIA2.0 showed the effect of LIAS on the pathological stages in KIRC and LUAD patients. **(D)** UALCAN website displayed the protein levels of LIAS in tumor and normal samples.

The expression profiles of LIAS were explored *via* TIMER2.0, GEPIA2.0, and UALCAN platforms. Firstly, we applied the TIMER2.0 platform to evaluate the LIAS expression profiles in both tumor tissues and normal tissues. We found that the LIAS expression was upregulated in some cancers, such as cholangiocarcinoma (CHOL), liver hepatocellular carcinoma (LIHC), LUAD, and lung squamous cell carcinoma (LUSC). Instead, the LIAS expression was downregulated in most cancers, like breast invasive carcinoma (BRCA), colon adenocarcinoma (COAD), KIRC, kidney renal papillary cell carcinoma (KIRP), prostate adenocarcinoma (PRAD), rectum adenocarcinoma (READ), thyroid carcinoma (THCA), and uterine corpus endometrial carcinoma (UCEC) (**Figure 1A**). Given that the TIMER2.0 database does not contain the peritumoral tissues in several kinds of cancers, GEPIA2.0 was used to explore the expression levels of LIAS between these tumors and corresponding normal tissues. The LIAS expression was significantly upregulated in lymphoid neoplasm diffuse large B-cell lymphoma (DLBC), brain lower-grade glioma (LGG), and thymoma (THYM). Conversely, the expression level of LIAS was downregulated in acute myeloid leukemia (LAML), OV, and testicular germ cell tumors (TGCT) (**Figure 1B**). However, the LIAS expression showed no obvious change in other cancers, including adrenocortical carcinoma (ACC), cervical squamous cell carcinoma and endocervical adenocarcinoma (CESC), glioblastoma multiforme (GBM), pancreatic adenocarcinoma (PAAD), pheochromocytoma and paraganglioma (PCPG), sarcoma (SARC), and uterine carcinosarcoma (UCS) (**Supplementary Figure S1**). Meanwhile, we employed the GEPIA2.0 database to analyze the correlation between the LIAS expression and the pathological stages. The violin plot conveyed that LIAS expression had a strong relationship with the stages of KIRC and LUAD patients (**Figure 1C**), whereas **Supplementary Figures S2A–U** showed that there existed no clear correlation between LIAS expression and pathological stages in other kinds of cancers. In addition, we analyzed the LIAS protein expression across cancers. We concluded that the protein levels of LIAS were downregulated in patients with OV and KIRC (**Figure 1D**).

**Figure 2:**
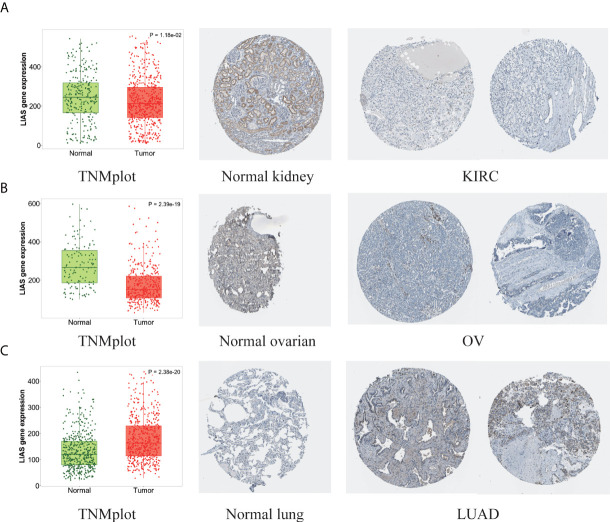
The different expression levels of LIAS in tumor and normal tissues. **(A–C)** TNMplot and HPA platforms displayed the downregulated expression of LIAS in KIRC and OV, and the upregulated expression in LUAD.

Next, the HPA database was employed to further confirm the protein levels of LIAS in cancers. LIAS showed weak or negative staining in KIRC and OV tissues and strong or medium staining in normal kidney and ovarian tissues. Moreover, LIAS showed weak or negative staining in normal lung tissues and strong or medium staining in the corresponding tumor tissues. In addition, TNMplot indicated the similar transcriptional changes of LIAS in KIRC, OV, and lung cancers (**Figures 2A–C**).

### The survival analysis of LIAS in cancers

**Figure 3:**
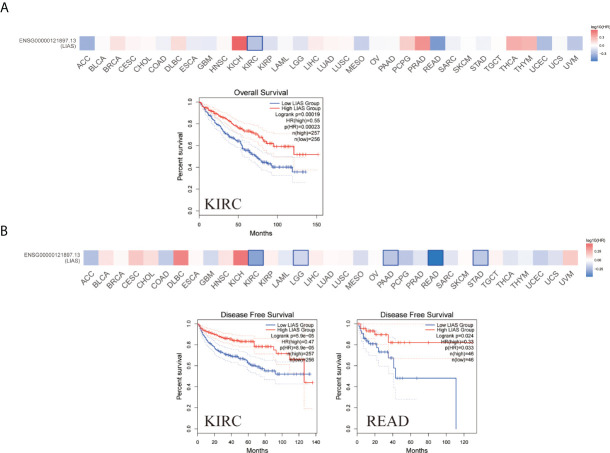
The prognostic values of LIAS across cancers. **(A, B)** GEPIA2.0 portrayed the effects of LIAS expression on the overall survival **(A)** and disease-free survival **(B)** in TCGA pan-cancer.

The GEPIA2.0 database was used to further investigate the prognostic values of LIAS expression in cancers. We divided the patients into high LIAS expression group and low LIAS expression group. A high LIAS expression was linked with the good OS in KIRC (p = 0.00019) (**Figure 3A**). Also, high expression levels of LIAS were related to the good DFS in KIRC (p = 5.9e-05) and READ (p = 0.024) (**Figure 3B**). Meanwhile, we applied the Kaplan–Meier plotter to evaluate the survival values of LIAS in breast cancer, ovarian cancer, and lung cancer. We concluded that breast cancer patients with a higher LIAS expression displayed better OS, distant metastasis-free survival (DMFS), post-progression survival (PPS), and relapse-free survival (RFS) (**Supplementary Figure S3A**). Moreover, ovarian cancer patients with a high LIAS expression displayed good OS, PFS, and PPS (**Supplementary Figure S3B**). The lung cancer patients with high expression levels of LIAS showed poor OS and first progression (FP) (**Supplementary Figure S3C**).

### The genetic alteration of LIAS in pan-cancer

**Figure 4:**
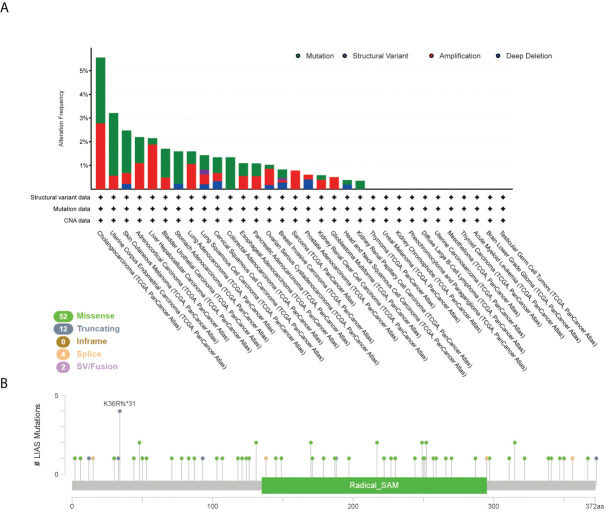
The mutation status of LIAS across TCGA cancers. **(A, B)** cBioPortal displayed the alteration frequency of mutation types and the mutation site on LIAS sequence.

Furthermore, we employed the cBioPortal tool to explore LIAS genetic alterations in pan-cancer. **Figure 4A** depicts that the amplification frequency of LIAS was the highest in CHOL. In addition, the mutation types and mutation sites within the LIAS sequence were explored. The mutation types of LIAS mainly contained missense mutation, truncating mutation, splice mutation, and fusion. Moreover, the missense mutation took up the major part of mutation types. The truncating mutation, K36Rf*31, might be a putative driver for cancers (**Figure 4B**).

**Figure 5:**
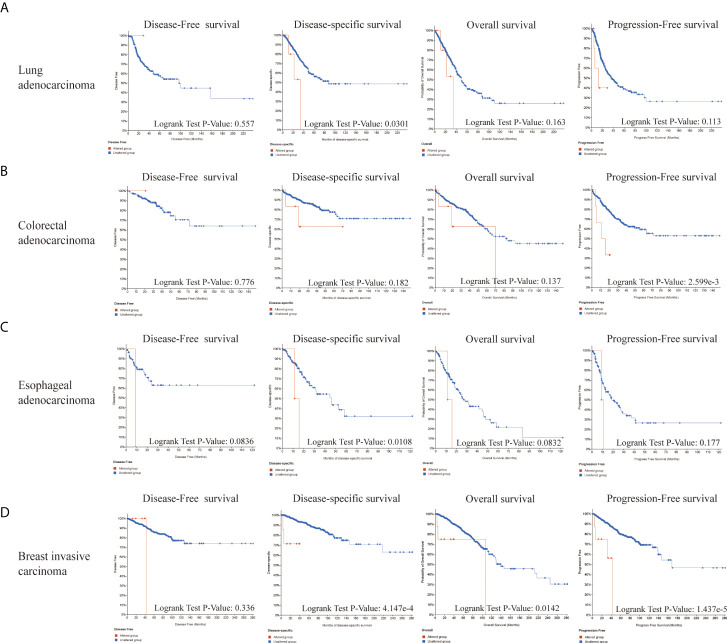
The prognosis analysis of LIAS alteration in cancers. **(A–D)** cBioPortal displayed the relationship between LIAS mutation and prognostic values (DSS, DFS, PFS, and OS) in some cancers, including lung adenocarcinoma, colorectal adenocarcinoma, esophageal adenocarcinoma, and breast invasive cancer.

Next, we explored the roles of LIAS genetic alterations on the patients’ prognosis. The LUAD patients with LIAS genetic alteration showed a poor prognosis in DSS (p = 0.0301), but not DFS (p = 0.557), OS (p = 0.163), and PFS (p = 0.113) (**Figure 5A**). Moreover, colorectal adenocarcinoma patients with LIAS genetic alteration displayed a poor prognosis in PFS (p = 2.599e-3), but not DFS (p = 0.776), DSS (p = 0.182), and OS (p = 0.137) (**Figure 5B**). Furthermore, esophageal adenocarcinoma (ESCA) patients with LIAS genetic alteration showed a poor prognosis in DSS (p = 0.0108), but not DFS (p = 0.0836), OS (p = 0.0832), and PFS (p = 0.177) (**Figure 5C**). Also, BRCA patients with LIAS genetic alteration illustrated a poor prognosis in DSS (p = 4.147e-4), OS (p = 0.0142), and PFS (p = 1.437e-5), but not DFS (p = 0.336) (**Figure 5D**). These findings demonstrated that the genetic alteration of LIAS exerted great effects on the prognostic values of above cancers.

### The methylation levels of LIAS in pan-cancer

**Figure 6:**
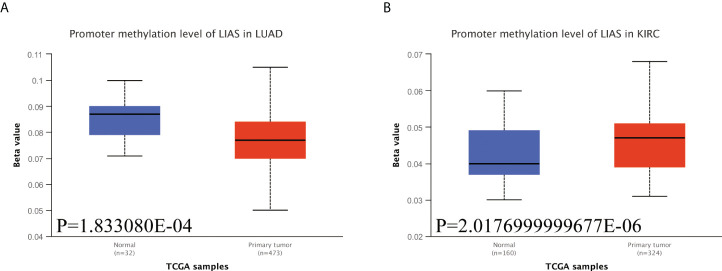
The methylation levels of LIAS in cancers. **(A, B)** The UALCAN database displayed the methylation levels of LIAS in patients with LUAD and KIRC, respectively.

Emerging studies have found that the alterations in DNA methylation patterns affect the expression profiles of cancer-associated genes (17, 18). Thus, we investigated the methylation levels of LIAS in TCGA pan-cancers by the UALCAN database. The diagraphs conveyed that the promoter methylation levels of LIAS in LUAD were lower than those in the normal group (**Figure 6A**). Moreover, the promoter methylation levels of LIAS in KIRC were higher than those in the normal group (**Figure 6B**), whereas there existed no significant changes in LIAS methylation levels in other cancers (**Supplementary Figures S4A–U**). These results indicated that the aberrantly expressed LIAS in LUAD and KIRC might be due to its promoter methylation.

### The effects of LIAS expression on the immune response

**Figure 7:**
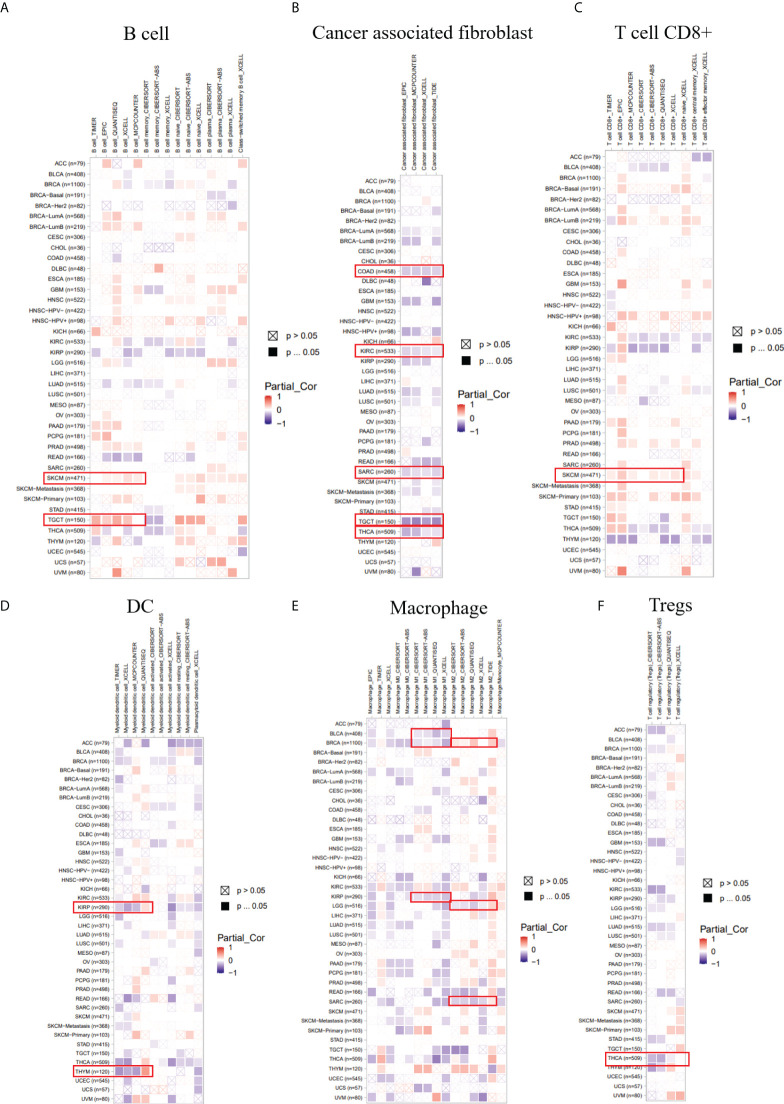
The correlation between the LIAS expression and several immune cells. **(A–F)** The TIMER2.0 database depicted the relationship between LIAS expression and immune infiltration of B cell **(A)**, cancer-associated fibroblast **(B)**, T cell CD8+ **(C)**, DC **(D)**, macrophage **(E)**, and Tregs **(F)** through several algorithms.

We employed several algorithms (TIMER (19), EPIC (20), QUANTISEQ (21), XCELL (22), MCPCOUNTER (23), CIBERSORT (24), and CIBERSORT-ABS) in the TIMER2.0 database to explore the correlation between immune infiltration cells and LIAS expression across TCGA cancers. We found that LIAS expression was positively related to the immune infiltration of B cells in SKCM and TGCT (**Figure 7A**). Moreover, LIAS expression had a negative relationship with cancer-associated fibroblast in COAD, KIRC, SARC, TGCT, and THCA (**Figure 7B**). The expression level of LIAS possessed a positive correlation with T-cell CD8+ in SKCM (**Figure 7C**). The LIAS expression was found to be negatively related to the immune infiltration of DC in KIRP and THYM (**Figure 7D**). Additionally, the expression level of LIAS was negatively correlated with M1 macrophage in BLCA, BRCA, and KIRP. The LIAS expression was positively related to M2 macrophage in BRCA and negatively related to M2 macrophage in LGG and SARC (**Figure 7E**). Meanwhile, the expression level of LIAS had a negative relationship with the immune infiltration of Tregs in THCA (**Figure 7F**). Nevertheless, there were no obvious correlations between LIAS expression and the immune infiltration of neutrophil, NK cell, and monocyte (**Supplementary Figures S5A–C**). The above findings portrayed the potential significance of LIAS in the immune infiltration of the tumor microenvironment.

We applied the TIDE database to explore the association between LIAS expression and several immune checkpoints in cancer patients. We found that, in bladder cancer patients treated with the PD-L1 inhibitor, LIAS expression was negatively associated with infiltration of cytotoxic T lymphocyte (CTL) (r = -0.203, p = 0.000142) (**Supplementary Figure S6A**). Moreover, in melanoma patients treated with the CTLA4 inhibitor, LIAS expression was negatively correlated with infiltration of CTL (r = -6.05e-01, p = 1.69e-02) (**Supplementary Figure S6B**). These findings revealed that the LIAS expression could predict the efficacy of immunotherapy in cancer patients. A higher expression level of LIAS could possess worse efficacy of immunotherapy, whereas more clinical trials are still needed to explore the correlation between LIAS expression and immunotherapy.

### The expression pattern of LIAS at a single-cell level

**Figure 8:**
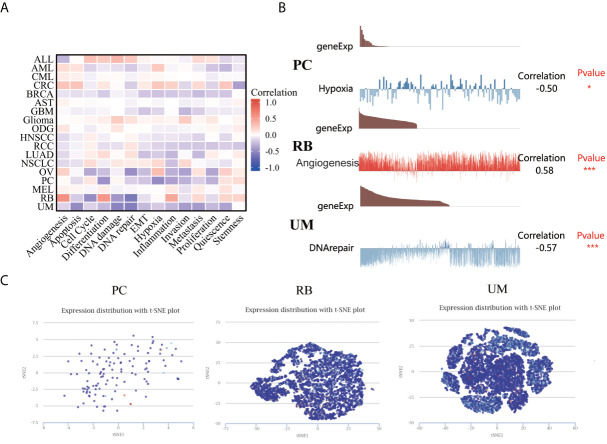
The expression levels of LIAS at a single-cell sequence level. **(A, B)** The CancerSEA tool displayed the relationship between LIAS expression and different functional statuses across pan-cancer. **(C)** The t-SNE diagrams portrayed the distributions of LIAS at single-cell levels from PC, RB, and UM samples.

The CancerSEA database was used to explore the expression levels of LIAS at a single-cell level and investigate the roles of LIAS in biological functional status. The LIAS expression in prostate cancer (PC) was negatively related to hypoxia and differentiation. The expression levels of LIAS in retinoblastoma (RB) were positively associated with angiogenesis and differentiation. Furthermore, LIAS expression in uveal melanoma (UM) had a negative relationship with DNA damage, DNA repair, and apoptosis (**Figure 8A**). At the same time, the pictures conveyed the correlation between the LIAS expression and hypoxia in PC, angiogenesis in RB, and DNA repair in UM (**Figure 8B**). Moreover, **Figure 8C** illustrates the expression distribution of LIAS in PC, RB, and UM at single-cell levels. From the above results, we could find that LIAS could be potentially crucial in the regulation of biological functions in cancers.

### Enrichment analysis of LIAS-related genes

**Figure 9:**
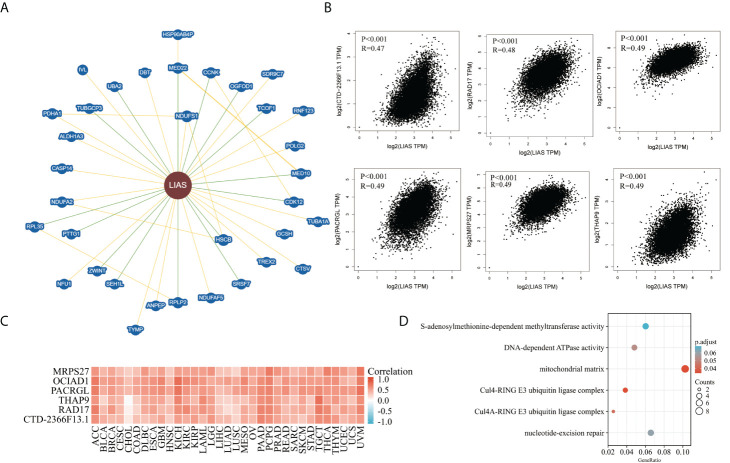
The functional enrichment analysis of LIAS-related genes across TCGA cancers. **(A)** The BioGRID database implicated the interactive network of LIAS-associated biomarkers. **(B)** GEPIA2.0 revealed that LIAS expression had a positive relationship with six genes (CTD-2366F13.1, RAD17, OCIAD1, PACRGL, MRPS27, and THAP9). **(C)** The heatmap revealed the positive correlation between LIAS expression and six genes (CTD-2366F13.1, RAD17, OCIAD1, PACRGL, MRPS27, and THAP9). **(D)** The G0/KEGG analysis of LIAS-related genes.

At last, we analyzed the functional enrichment of the LIAS-related genes in cancers. BioGRID was applied to explore the LIAS-interacted biomarkers (**Figure 9A**). Next, the GEPIA2.0 database was used to download the top 100 LIAS-associated genes **(** **Supplementary Table S1** **)**. The expression levels of LIAS had a positive correlation with the expression levels of CTD-2366F13.1 (R = 0.47, p < 0.001), RAD17 (R = 0.48, p < 0.001), OCIAD1 (R = 0.49, p < 0.001), PACRGL (R = 0.49, p < 0.001), MRPS27 (R = 0.49, p < 0.001), and THAP9 (R = 0.49, p < 0.001) in pan-cancer (**Figure 9B**). Afterward, the heatmap portrayed that LIAS was positively related to the above genes across most cancers (**Figure 9C**). The GO and KEGG enrichment illustrated that LIAS-related genes were mostly enriched in some cancer-associated pathways, including mitochondrial matrix and nucleotide-excision repair (**Figure 9D**).

## Discussion

A recent study has reported that LIAS was highly expressed in bladder cancer tissues (25). However, the underlying mechanisms of LIAS in cancers should be further explored and elucidated. In order to explore the significant role of LIAS in different kinds of cancers, several bioinformatics platforms were employed to carry out a comprehensive analysis of LIAS in pan-cancer. We investigated the LIAS expression in 33 kinds of cancers based on TCGA database. Moreover, the LIAS expression was upregulated in some cancers, such as CHOL, LIHC, LUAD, and LUSC. On the contrary, the expression levels of LIAS were downregulated in BRCA, COAD, KIRC, KIRP, PRAD, READ, THCA, and UCEC. Then, the HPA database portrayed that LIAS displayed weak or negative staining in KIRC and OV tissues and strong or medium staining in LUAD tissues. Meanwhile, the methylation level of LIAS in the LUAD group was lower than in the corresponding normal group. The methylation level of LIAS was higher in the KIRC group than in the corresponding normal group. Moreover, GEPIA2.0 further implicated that high LIAS expression was related to good OS in KIRC, DFS in KIRC, and READ. At the same time, the Kaplan–Meier plotter was applied to find that breast cancer patients with a high LIAS expression showed good OS, DMFS, PPS, and RFS. Ovarian cancer patients with high LIAS expression showed good OS, PFS, and PPS. Also, lung cancer patients with higher LIAS expression showed worse OS and FP. These results have demonstrated that aberrant LIAS could be a potential prognostic predictive molecule for cancers.

Innate immune cells could trigger the adaptive immune responses, and understanding of cancer-intrinsic processes will supply the exploration and development of immunotherapy (26, 27). Cancer-associated fibroblast was reported to induce cancer cell proliferation, invasion, and metastasis through various pathways (28, 29). Moreover, Tregs that expressed the transcription factor Foxp3 could possess importance in immune homeostasis (26, 30). In this study, we found that LIAS expression was strongly correlated with the infiltration of immune cells, including B cell, cancer-associated fibroblast, T cell CD8+, DC, macrophage, and Tregs. A study has demonstrated that immune checkpoint blockade could exert great effects on the cancer therapy through inhibiting the suppression of T-cell activation (31). The inhibitors of PD-L1 and PD-1 have been verified to possess promising therapeutic values in most cancers (32). Another study has clarified that immune checkpoint blockade (ICB) could be beneficial to the progress of cancer therapy (33, 34). In this article, we concluded that in bladder cancer patients treated with the PD-L1 inhibitor, LIAS expression was negatively associated with infiltration of CTL. Moreover, in melanoma patients treated with the CTLA4 inhibitor, LIAS expression was negatively linked with infiltration of CTL.

In conclusion, we explored the expression levels, survival analysis, methylation levels, genetic alteration, and immune analysis of LIAS in pan-cancer by comprehensive bioinformatics technologies. Furthermore, the LIAS expression at a single-cell sequencing level and its roles in the regulation of signaling pathways were investigated. The above results have implicated that LIAS could be a novel biomarker for the prognostic prediction and immune response in several cancer patients.

## Data availability statement

The original contributions presented in the study are included in the article/**Supplementary Material**. Further inquiries can be directed to the corresponding author/s.

## Author contributions

YC and QCH: acquisition of data. WL, QL and BP: analysis and interpretation of data. JP and ZX: conception and design. JL and WZ: data curation. FK, QHH, and YY: development of methodology. Cai Y, Xu Z, and Bai N: writing of the manuscript and revision of the manuscript. All authors contributed to the article and approved the submitted version.

## Funding

This study is supported by grants from the Natural Science Foundation of Hunan Province (2021JJ30904) and the horizontal project (2021-021, 1 43010100).

## Conflict of interest

The authors declare that the research was conducted in the absence of any commercial or financial relationships that could be construed as a potential conflict of interest.

## Publisher’s note

All claims expressed in this article are solely those of the authors and do not necessarily represent those of their affiliated organizations, or those of the publisher, the editors and the reviewers. Any product that may be evaluated in this article, or claim that may be made by its manufacturer, is not guaranteed or endorsed by the publisher.

## Supplementary material

Supplementary Figure 1 GEPIA2.0 conveyed the LIAS expression between tumor tissues and normal tissues.

Supplementary Figure 2 The effects of LIAS expression on the pathological stages in pan-cancer. **(A–U)** GEPIA2.0 portrayed the effects of LIAS expression on the pathological stages of cancer patients, including ACC, BLCA, BRCA, CESC, CHOL, COAD, DLBC, ESCA, HNSC, KICH, KIRP, LIHC, LUSC, READ, OV, SKCM, STAD, TGCT, THCA, UCEC and PAAD.

Supplementary Figure 3 The prognostic values of LIAS expression in three kinds of cancers. **(A–C)** The Kaplan-Meier plotter displayed the effects of LIAS expression on the prognostic values (OS, DMFS, PPS, RFS, PFS and FP) in three cancers, including breast cancer, ovarian cancer and lung cancer.

Supplementary Figure 4 The methylation levels of LIAS in several cancers. **(A–U)** The UALCAN database revealed the methylation levels of LIAS in CESC, CHOL, COAD, KIRP, LIHC, PCPG, READ, STAD, THYM, BLCA, BRCA, ESCA, LUSC, PAAD, PRAD, TGCT, THCA, UCEC, HNSC, GBM and SARC, respectively.

Supplementary Figure 5 The correlation between expression levels of LIAS and immune cell infiltration. **(A–C)** The TIMER2.0 database the relationship between LIAS expression and immune infiltration of neutrophil, NK cell and monocyte.

Supplementary Figure 6 The expression levels of LIAS and immune checkpoints. **(A, B)** TIDE database explored the relationship between LIAS expression and infiltration of CTL in cancer patients, including bladder cancer patients treated with PD-L1 inhibitor and melanoma patients treated with CTLA4 inhibitor.
