# Supplementary figures and images for: Comprehensive analysis of the potential cuproptosis-related biomarker LIAS that regulates prognosis and immunotherapy of pan-cancers
Source: Front Oncol. 2022 Aug 2;12:952129. doi: 10.3389/fonc.2022.952129 (PMC9379260; doi:10.3389/fonc.2022.952129)

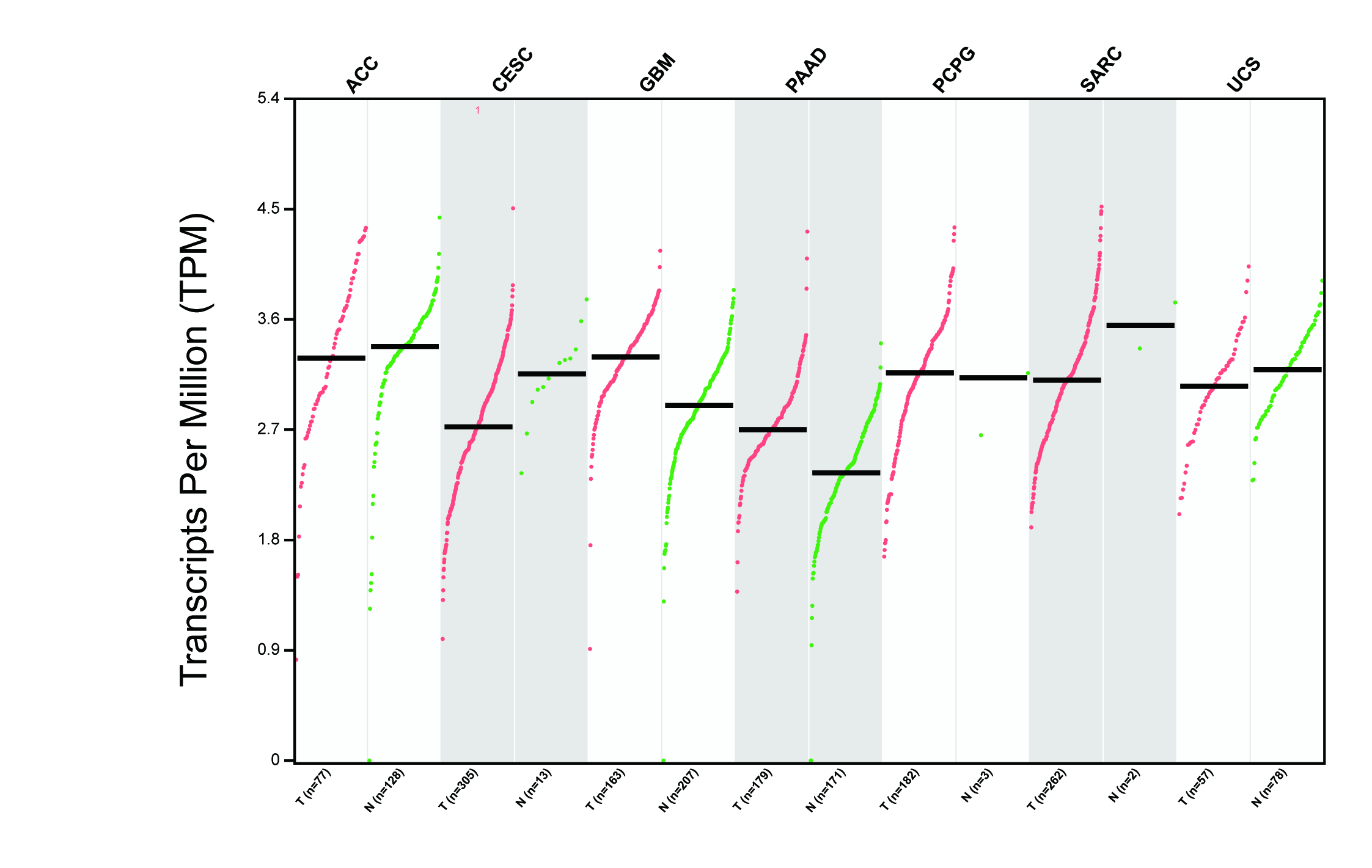

Supplement: Supplementary Figure 1 — GEPIA2.0 conveyed the LIAS expression between tumor tissues and normal tissues. [file Image_1.tif]

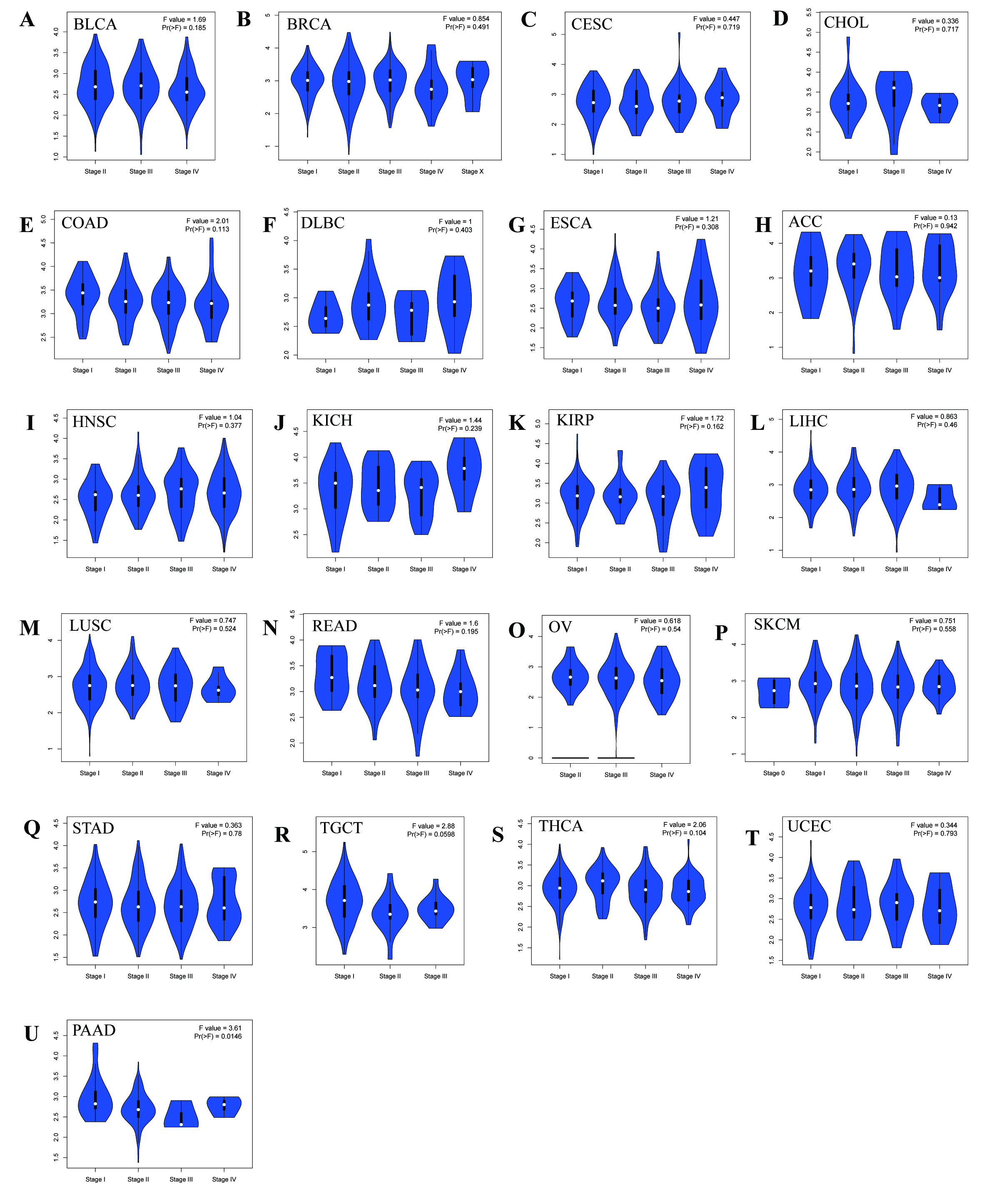

Supplement: Supplementary Figure 2 — The effects of LIAS expression on the pathological stages in pan-cancer. (A–U) GEPIA2.0 portrayed the effects of LIAS expression on the pathological stages of cancer patients, including ACC, BLCA, BRCA, CESC, CHOL, COAD, DLBC, ESCA, HNSC, KICH, KIRP, LIHC, LUSC, READ, OV, SKCM, STAD, TGCT, THCA, UCEC and PAAD. [file Image_2.tif]

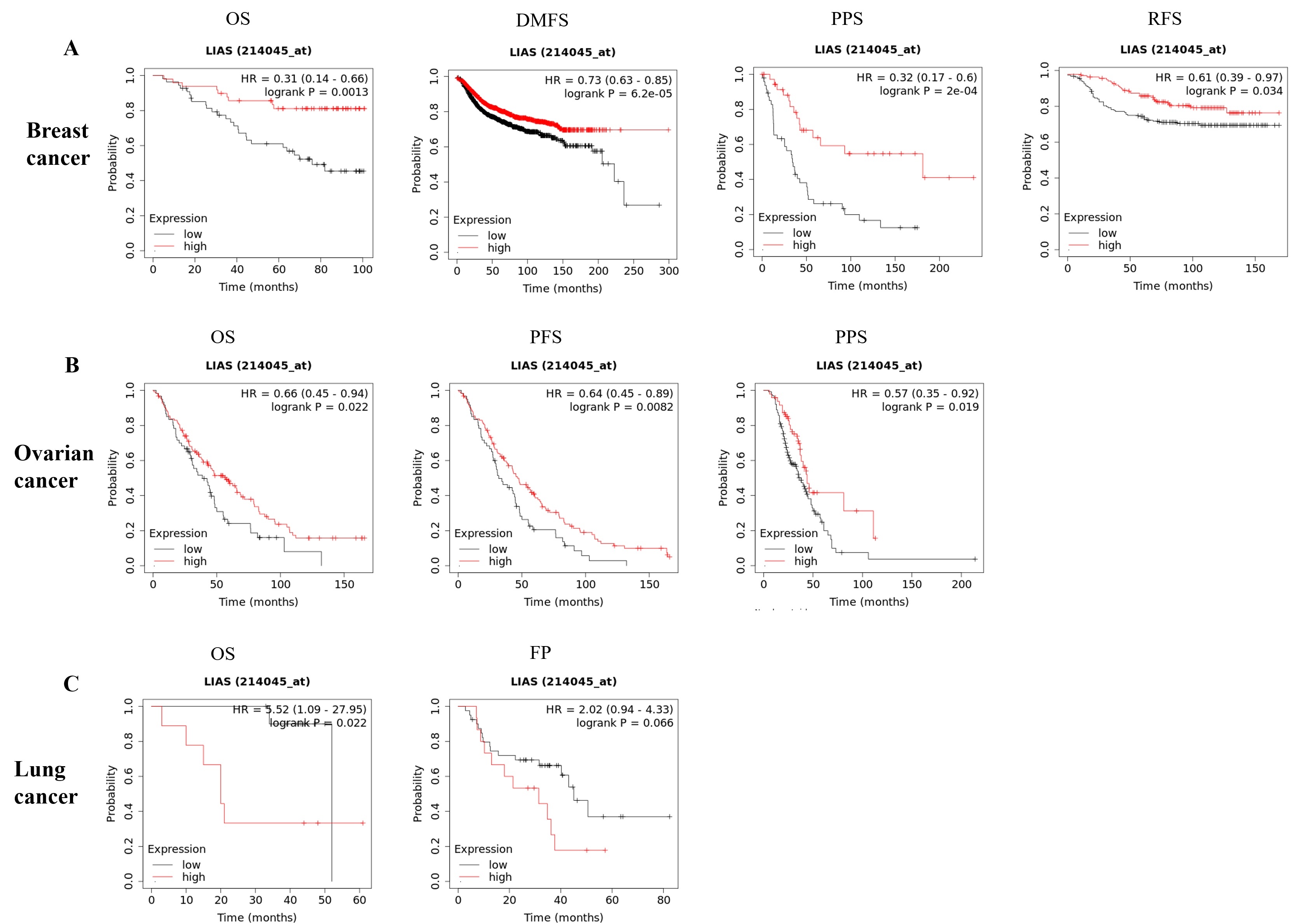

Supplement: Supplementary Figure 3 — The prognostic values of LIAS expression in three kinds of cancers. (A–C) The Kaplan-Meier plotter displayed the effects of LIAS expression on the prognostic values (OS, DMFS, PPS, RFS, PFS and FP) in three cancers, including breast cancer, ovarian cancer and lung cancer. [file Image_3.tif]

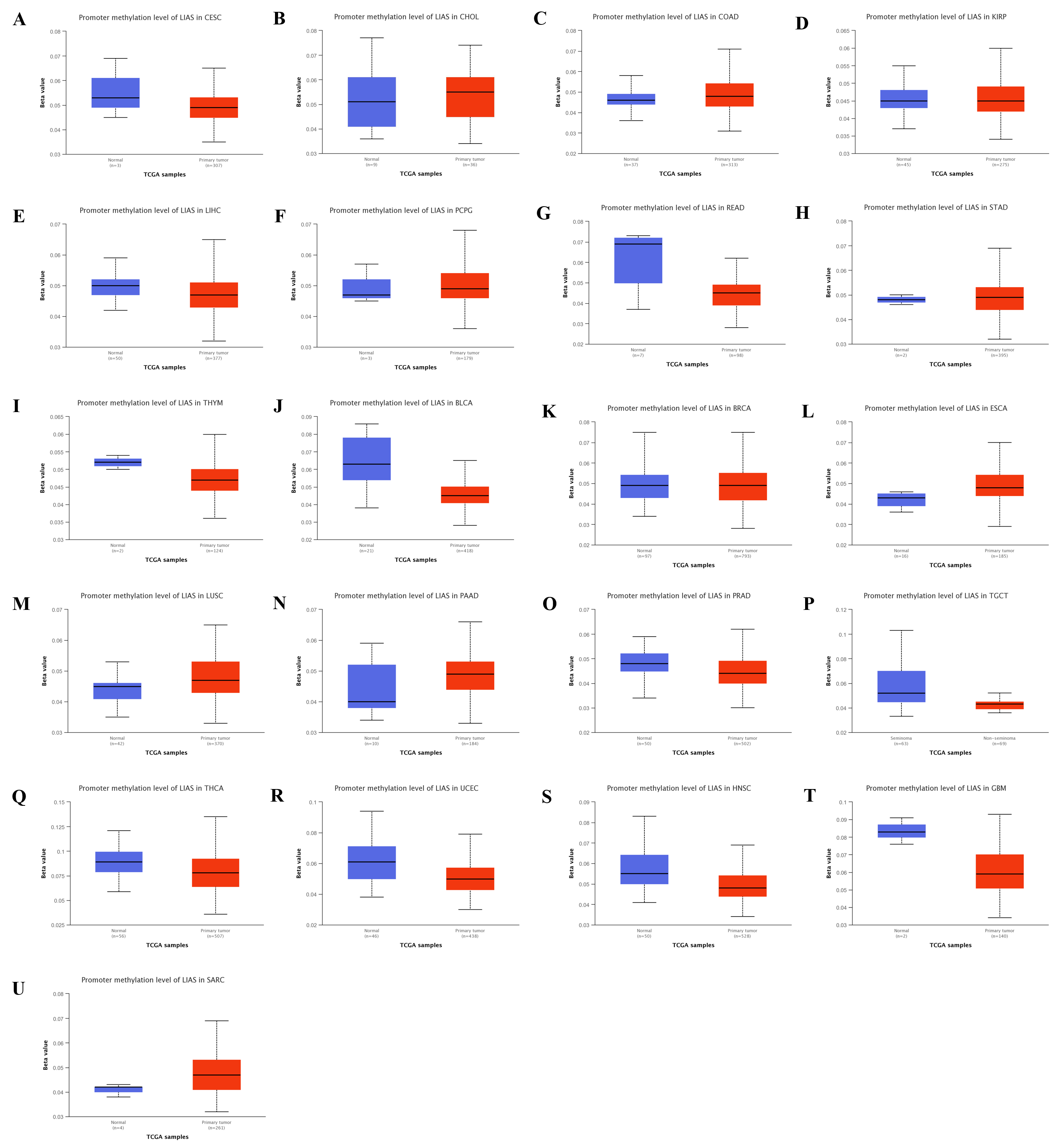

Supplement: Supplementary Figure 4 — The methylation levels of LIAS in several cancers. (A–U) The UALCAN database revealed the methylation levels of LIAS in CESC, CHOL, COAD, KIRP, LIHC, PCPG, READ, STAD, THYM, BLCA, BRCA, ESCA, LUSC, PAAD, PRAD, TGCT, THCA, UCEC, HNSC, GBM and SARC, respectively. [file Image_4.tif]

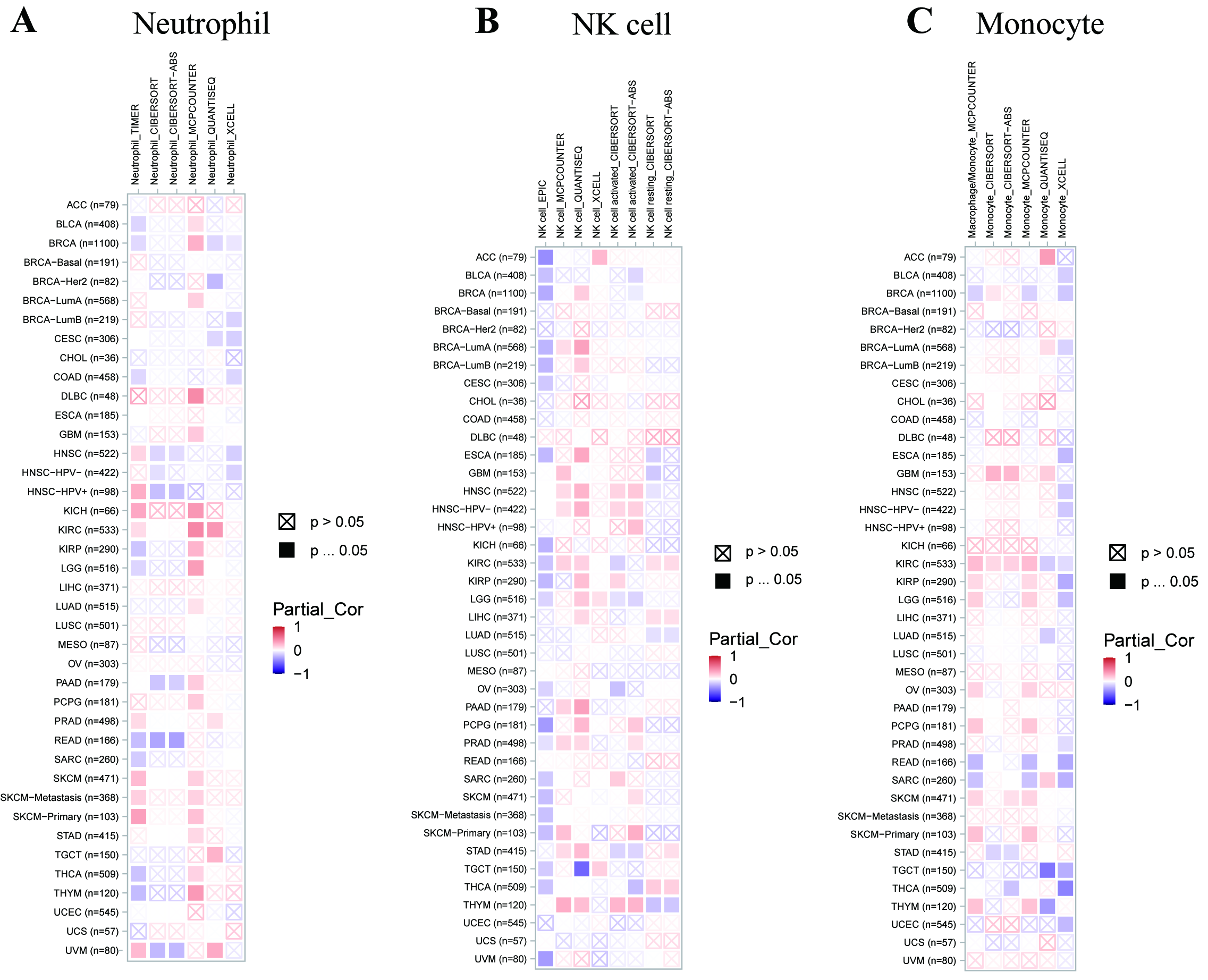

Supplement: Supplementary Figure 5 — The correlation between expression levels of LIAS and immune cell infiltration. (A–C) The TIMER2.0 database the relationship between LIAS expression and immune infiltration of neutrophil, NK cell and monocyte. [file Image_5.tif]

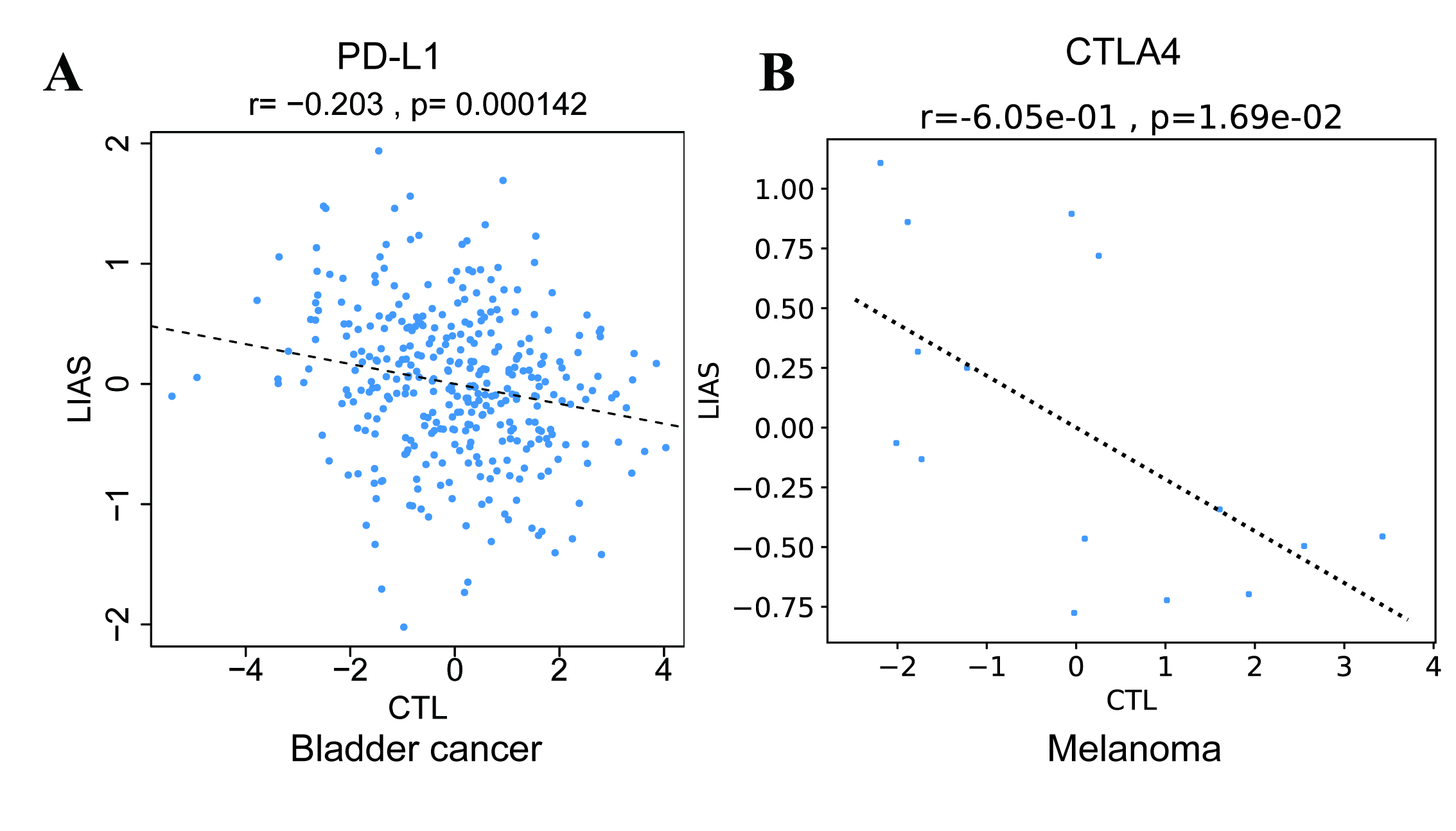

Supplement: Supplementary Figure 6 — The expression levels of LIAS and immune checkpoints. (A, B) TIDE database explored the relationship between LIAS expression and infiltration of CTL in cancer patients, including bladder cancer patients treated with PD-L1 inhibitor and melanoma patients treated with CTLA4 inhibitor. [file Image_6.tif]
