# Supplementary material for: Comprehensive analysis of the potential cuproptosis-related biomarker LIAS that regulates prognosis and immunotherapy of pan-cancers
Source: Front Oncol. 2022 Aug 2;12:952129. doi: 10.3389/fonc.2022.952129 (PMC9379260; doi:10.3389/fonc.2022.952129)
Supplement: Supplementary file 7 [file Table_1.docx]

| **Gene Symbol**  **Table 1. The top 100 LIAS-related genes that obtained from the GEPIA2.0 database.** | **Gene ID** | **PCC** |
| --- | --- | --- |
| MRPS27 | ENSG00000113048.16 | 0.49 |
| OCIAD1 | ENSG00000109180.14 | 0.49 |
| PACRGL | ENSG00000163138.18 | 0.49 |
| THAP9 | ENSG00000168152.12 | 0.49 |
| RAD17 | ENSG00000152942.18 | 0.48 |
| CTD-2366F13.1 | ENSG00000247796.2 | 0.47 |
| ZCCHC4 | ENSG00000168228.14 | 0.47 |
| LETMD1 | ENSG00000050426.15 | 0.47 |
| RFC1 | ENSG00000035928.14 | 0.47 |
| TMEM128 | ENSG00000132406.11 | 0.46 |
| OSGEPL1 | ENSG00000128694.11 | 0.46 |
| ADAL | ENSG00000168803.14 | 0.46 |
| KIAA0232 | ENSG00000170871.11 | 0.46 |
| ZNF721 | ENSG00000182903.15 | 0.45 |
| COX15 | ENSG00000014919.12 | 0.45 |
| SLC30A9 | ENSG00000014824.13 | 0.44 |
| FAN1 | ENSG00000198690.9 | 0.44 |
| STX18-AS1 | ENSG00000247708.7 | 0.44 |
| MRFAP1L1 | ENSG00000178988.10 | 0.43 |
| LINC00909 | ENSG00000264247.1 | 0.43 |
| RAB28 | ENSG00000157869.14 | 0.43 |
| PMPCB | ENSG00000105819.13 | 0.43 |
| GNPDA2 | ENSG00000163281.11 | 0.43 |
| KDM3B | ENSG00000120733.13 | 0.43 |
| ZBTB49 | ENSG00000168826.15 | 0.43 |
| CRYZL1 | ENSG00000205758.11 | 0.43 |
| BRD8 | ENSG00000112983.17 | 0.43 |
| COQ5 | ENSG00000110871.14 | 0.42 |
| TATDN3 | ENSG00000203705.10 | 0.42 |
| LYRM7 | ENSG00000186687.15 | 0.42 |
| C17orf80 | ENSG00000141219.15 | 0.42 |
| CRBN | ENSG00000113851.13 | 0.42 |
| RP11-284M14.1 | ENSG00000250326.1 | 0.42 |
| PTCD2 | ENSG00000049883.14 | 0.42 |
| PEX12 | ENSG00000108733.9 | 0.42 |
| SEPSECS | ENSG00000109618.11 | 0.42 |
| PPIL3 | ENSG00000240344.8 | 0.42 |
| CBR4 | ENSG00000145439.11 | 0.42 |
| CTC-559E9.1 | ENSG00000266904.5 | 0.42 |
| DNAJC27 | ENSG00000115137.11 | 0.42 |
| FAM175A | ENSG00000163322.13 | 0.42 |
| DIS3L | ENSG00000166938.12 | 0.42 |
| METAP1D | ENSG00000172878.13 | 0.41 |
| DCAF16 | ENSG00000163257.10 | 0.41 |
| MATR3 | ENSG00000015479.17 | 0.41 |
| TMEM161B | ENSG00000164180.13 | 0.41 |
| AASDH | ENSG00000157426.13 | 0.41 |
| AGGF1 | ENSG00000164252.12 | 0.41 |
| MRFAP1 | ENSG00000179010.14 | 0.41 |
| GFM2 | ENSG00000164347.17 | 0.41 |
| UNC119B | ENSG00000175970.8 | 0.41 |
| ATG14 | ENSG00000126775.8 | 0.41 |
| ERCC8 | ENSG00000049167.13 | 0.41 |
| BOD1L1 | ENSG00000038219.12 | 0.41 |
| GUF1 | ENSG00000151806.13 | 0.4 |
| MBLAC2 | ENSG00000176055.9 | 0.4 |
| ZFP2 | ENSG00000198939.7 | 0.4 |
| YTHDC1 | ENSG00000083896.12 | 0.4 |
| PAIP2 | ENSG00000120727.12 | 0.4 |
| GIN1 | ENSG00000145723.16 | 0.4 |
| LARP7 | ENSG00000174720.15 | 0.4 |
| EZH1 | ENSG00000108799.12 | 0.4 |
| N6AMT1 | ENSG00000156239.11 | 0.4 |
| SENP8 | ENSG00000166192.14 | 0.4 |
| COPS4 | ENSG00000138663.8 | 0.4 |
| KIAA1109 | ENSG00000138688.15 | 0.4 |
| GPBP1 | ENSG00000062194.15 | 0.4 |
| RP11-522I20.3 | ENSG00000254473.1 | 0.4 |
| TAPT1 | ENSG00000169762.16 | 0.4 |
| LARS | ENSG00000133706.17 | 0.4 |
| RP11-472B18.1 | ENSG00000224097.5 | 0.4 |
| TRIM23 | ENSG00000113595.14 | 0.4 |
| C9orf156 | ENSG00000136932.13 | 0.4 |
| MTMR10 | ENSG00000166912.16 | 0.4 |
| TMEM170A | ENSG00000166822.12 | 0.4 |
| USP30 | ENSG00000135093.12 | 0.4 |
| DZIP3 | ENSG00000198919.12 | 0.4 |
| HELQ | ENSG00000163312.10 | 0.39 |
| TRAM2-AS1 | ENSG00000225791.6 | 0.39 |
| ZNF658B | ENSG00000198416.9 | 0.39 |
| KCTD7 | ENSG00000243335.8 | 0.39 |
| ZNF554 | ENSG00000172006.11 | 0.39 |
| DPH5 | ENSG00000117543.19 | 0.39 |
| FAM200A | ENSG00000221909.2 | 0.39 |
| ZNF555 | ENSG00000186300.11 | 0.39 |
| SPATA7 | ENSG00000042317.16 | 0.39 |
| RP11-77K12.9 | ENSG00000274220.1 | 0.39 |
| ANKRA2 | ENSG00000164331.9 | 0.39 |
| CTD-2017C7.2 | ENSG00000259088.1 | 0.39 |
| MAP2K5 | ENSG00000137764.19 | 0.39 |
| RP11-121C2.2 | ENSG00000259959.1 | 0.39 |
| WDR48 | ENSG00000114742.13 | 0.39 |
| NUDT6 | ENSG00000170917.13 | 0.39 |
| SMARCAD1 | ENSG00000163104.17 | 0.39 |
| RP11-73K9.2 | ENSG00000260526.1 | 0.39 |
| ZNF84 | ENSG00000198040.10 | 0.39 |
| RP11-539G18.3 | ENSG00000255458.5 | 0.39 |
| ZDHHC17 | ENSG00000186908.14 | 0.39 |
| EIF4ENIF1 | ENSG00000184708.17 | 0.39 |
| ZNF23 | ENSG00000167377.17 | 0.39 |
